# Relationship Between Radiological Features of Primary Empty or Primary Partial Empty Sella and Pituitary Hormone Levels

**DOI:** 10.3390/biomedicines13030722

**Published:** 2025-03-15

**Authors:** Bernadetta Kałuża, Mariusz Furmanek, Jan Domański, Aleksandra Żuk-Łapan, Emilia Babula, Iga Poprawa, Jerzy Walecki, Edward Franek

**Affiliations:** 1 Department of Internal Medicine, Endocrinology and Diabetology, National Medical Institute of the Ministry of the Interior and Administration in Warsaw, 02-507 Warsaw, Polandebabula99@gmail.com (E.B.);; 2 Students Scientific Group of the Medical University of Warsaw at the Department of Internal Medicine, Endocrinology and Diabetology, National Medical Institute of the Ministry of the Interior and Administration in Warsaw, 02-507 Warsaw, Poland; 3 Department of Radiology, National Institute of Medicine of the Ministry of the Interior and Administration, 02-507 Warsaw, Poland; 4 Department of Pediatric Radiology, Medical University of Warsaw, 02-091 Warsaw, Poland; 5 Department of Human Epigenetics, Mossakowski Medical Research Centre, Polish Academy of Sciences, 02-106 Warsaw, Poland

**Keywords:** empty sella, partial empty sella

## Abstract

**Purpose:** The purpose of this study was to assess the relationship between the radiological criteria determining an primary empty or primary partial empty sella and the pituitary hormone levels. **Methods**: Out of 594 patients who underwent pituitary magnetic resonance imaging (MRI), we selected 43 patients with primarily empty and partial empty sella and conducted a prospective evaluation of pituitary MRI in 2022. Pituitary craniocaudal (CC) diameter, pituitary volume, sellar volume, pituitary volume expressed as a percentage of sellar volume (bony sella), and pituitary height expressed as a percentage of sellar height (craniocaudal) were assessed. Serum pituitary hormone concentrations were measured, and a logistic regression analysis was performed to assess a relationship between the radiological and hormonal parameters. **Results**: Only six patients (14%) exhibited abnormal hormone levels. None of the assessed radiological parameters were correlated with the presence of the hormonal disorders either in the univariate or multivariate logistic regression analysis. The univariate logistic regression analysis revealed a significant relationship between age and the hormonal disorders (OR 0.916 [0.844–0.993]; *p* = 0.034), but this was not confirmed in the multivariate analysis. **Conclusions**: These findings suggest that radiological parameters alone are insufficient to predict hormonal dysfunction in patients with empty or partial empty sella. However, younger patients may be at a higher risk, warranting closer hormonal monitoring.

## 1. Introduction

Located in the middle cranial fossa, the sella turcica is formed by the middle part of the superior surface of the sphenoid body, which is posterior to the tuberculum sellae and prechiasmatic sulcus, anterior to the dorsum sellae, and medial to the carotid sulcus on either side. An indentation in the sella turcica, known as the hypophyseal fossa, houses the pituitary gland [1,2,3]. The anterior lobe of the pituitary (adenohypophysis) is composed of cell lines producing hormones (growth hormone, prolactin, adrencorticotropic hormone, thyroid-stimulating hormone, luteinizing hormone, and follicle-stimulating hormone), whereas its posterior lobe (neurohypophysis) stores and releases oxytocin and antidiuretic hormone [4]. The formation of the pituitary gland from the Rathke’s pouch and from an elevation in the floor of the third ventricle precedes the formation of the sella turcica and thus affects its structure. As a result, the close relationship between these two structures begins during embryogenesis. Sellar enlargement may be associated with pituitary pathologies, such as adenomas or cysts; conversely, a reduced sellar size may be associated with primary hypopituitarism or growth hormone deficiency [4,5].

An empty or partial empty sella results from the subarachnoid space becoming herniated into the sella turcica and compressing the pituitary gland [6]. This condition may be caused by congenital absence or weakening of the diaphragma sellae due to changes in intracranial pressure or pituitary volume (e.g., during pregnancy). Such conditions are classified as a primary empty or partial empty sella [6,7,8,9]. In contrast, a secondary empty or partial empty sella may be due to a neurosurgical intervention, cranial injury, pituitary apoplexy, tumor, hyperplasia, or inflammation [6,7,8,9]. The prevalence of empty sella based on pituitary magnetic resonance imaging (MRI) or on clinical criteria varies in various reports, ranging from 2% to 35% [10,11,12]. Up to 79% of empty sella diagnoses are incidental, with as many as 50% of these patients exhibiting hypopituitarism [4]. An empty sella has been revealed in 5.5–12% of postmortem examinations and in up to approximately 12% of imaging studies, while its prevalence in patients with idiopathic intracranial hypertension may reach 70–94% [4,10]. The discrepancies in the reported rates of empty or partial empty sellae may be due to adopting various criteria in defining the condition [7]. An empty sella is usually defined as a herniation of the subarachnoid space into the hypophyseal fossa to take up more than 50% of its volume and flattening the pituitary to less than 3 mm craniocaudally, whereas a partial empty sella is characterized by the pituitary measuring at least 3 mm craniocaudally, with less than 50% of the sella volume filled with the cerebrospinal fluid [7,9,13,14,15]. The discrepancies in the reported prevalence of empty sella may be due to the adopted definition using a fixed cutoff pituitary height or proportion of the pituitary–sella volume; alternatively, they may be due to the varying resolution of the scans, which has changed over time. Pituitary height (i.e., its CC diameter) is not the only radiological feature of empty or partial empty sella. The effects of the suprasellar cistern compression, such as pituitary flattening, infundibular displacement or bending, and sellar volume enlargement, are not only additional radiological features of empty or partial empty sella but may also be predictors of endocrine disorders [16,17]. Apart from these established radiological features, there are also others that are not commonly considered, such as pituitary anteroposterior (AP) and transverse diameters, pituitary volume, sellar diameters, sellar volume, and correlations between pituitary volume and sellar volume.

The purpose of this study was to radiologically assess patients with empty and partial empty sella and to assess the relationship between the radiological criteria that define an empty or partial empty sella and hormonal disorders—not only by determining the prevalence of these phenomena but also by calculating the risk of endocrine disorders in this patient population.

## 2. Material and Methods

**Figure 1:**
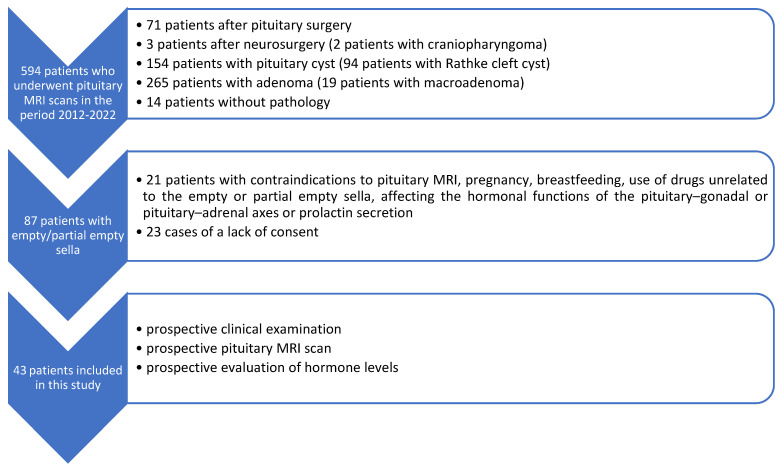
Flow diagram presenting the process of collecting the data included in this study.

In order to conduct this study, we reviewed data from the Department of Diagnostic Radiology (National Medical Institute of the Ministry of the Interior and Administration, Warsaw, Poland) database from the years 2012–2022. We found 594 cases of patients who underwent pituitary MRI scans in order to visualize (a) primary or secondary empty or partial empty sella previously detected with MRI of the central nervous system; (b) status post neurosurgery on the central nervous system, including craniopharyngioma surgery; (c) status post pituitary surgery; (d) Rathke’s cleft cyst; and (e) pituitary adenoma (Figure 1). Eighty-seven of those patients exhibited morphological features of an empty or partial empty sella. These 87 patients were then assessed prospectively in terms of study inclusion criteria. The study inclusion criteria were age over 18 years and primary empty or primary partial empty sella in pituitary MRI. The study exclusion criteria were history of surgical interventions or irradiation of the central nervous system, a tumor of the pituitary gland or central nervous system, pregnancy, breastfeeding, the use of medications unconnected with empty or partial empty sella affecting endocrine function of the pituitary–gonadal or pituitary–adrenal axes or prolactin secretion, contraindications to pituitary MRI, and a lack of patient’s consent.

Out of the patients with primary empty or partial empty sella, we ultimately included 43 patients who underwent another prospective pituitary MRI scan performed by an experienced radiologist, the same one each time. The time interval between the previous and the current pituitary MRI scans was 3.651 ± 1.675 years; there were no differences between these scans. Based on the existing definitions, we defined an empty sella as a herniation of the subarachnoid space into the sella turcica exceeding 50% of its volume and leading to the flattening of the pituitary to a craniocaudal diameter of less than 3 mm, whereas partial empty sella was diagnosed when the craniocaudal diameter of the pituitary was greater than or equal to 3 mm and less than 50% of the sella turcica volume was occupied by the cerebrospinal fluid [9,13,14,15,17].

Each patient underwent pituitary and optic nerve MRI at the Department of Diagnostic Radiology with the use of a 3.0 Tesla scanner, with a slice thickness of 2 mm, slice gap of 0, field of view 150/150, matrix 240/300, and pixel alimentation of 0.5 × 0.625 mm. A pituitary protocol was used, with T1-weighted (+Dixon), T2-weighted, FLAIR, T2 STIR, and contrast-enhanced T1-weighted sequences being obtained. The AP, coronal (right–left, RL), and CC diameters of the pituitary, the sella turcica, and the optic nerve sheath were measured. Tortuous optic nerves, enlarged cerebrospinal fluid spaces, and critical stenosis of the transverse or sigmoid sinus were taken into consideration. The pituitary volume was calculated manually based on a simplified ellipsoid formula (volume = 0.5 × AP × RL × CC). The sellar volume was calculated based on a simplified ellipsoid formula (volume = 0.5 × AP × RL × CC). The craniocaudal diameter of the sella turcica was based either on the bony landmarks or, alternatively, only on the location of the diaphragma sellae. Taking into account previous diagnoses and current medications, the patients included in this study underwent hormone profile assessments, fasting blood tests in the morning at about 8:00 a.m. after sleeping through the night, and a urine test from a morning sample. In order to assess anterior pituitary function, adrenocorticotropic hormone (ACTH), cortisol, growth hormone, insulin-like growth factor 1 (IGF-1), prolactin, thyroid-stimulating hormone (TSH), free triiodothyronine (fT3), free thyroxine (fT4), follicle-stimulating hormone (FSH), luteinizing hormone (LH), estradiol, and testosterone levels were measured; to indirectly assess posterior pituitary function, urine-specific gravity, serum osmolality, urine osmolality, and sodium and potassium levels were measured.

Previously diagnosed hormonal disorders due to empty or partial empty sella were taken into account during clinical examination in accordance with current standards [18]. Hyperprolactinemia was diagnosed when routine blood samples (morning fasting, after a night of regular sleep, and after a rest) showed elevated prolactin levels or when such a finding had been observed on previous clinical assessments and other potential causes (e.g., stress or medication) had been excluded. Diagnosis of secondary adrenocortical insufficiency was based on low ACTH and cortisol levels in conjunction with manifestations of adrenocortical insufficiency (orthostatic hypotension, syncope, nausea, vomiting, anorexia, weight loss, skin hypopigmentation, and hypoglycemia) and, in case of any uncertainties, was additionally confirmed with synthetic corticotropin-releasing hormone tests. The criteria adopted for diagnosing secondary hypothyroidism were low TSH levels together with low fT3 and fT4 levels and associated symptoms (hair loss, dry skin, cold intolerance, psychomotor retardation, constipation, bradycardia, hoarseness, anemia, or hyperlipidemia), and uncertain cases were decided with a thyrotropin-releasing hormone test. Growth hormone deficiency was diagnosed based on the following criteria: low IGF-1 levels in conjunction with symptoms (hypoglycemia, hyperlipidemia, weakness, low muscle mass, abdominal obesity, and low bone mineral density) or a test with insulin. The criteria adopted for determining sex hormone deficiency were low LH and FSH levels in conjunction with low estradiol levels and symptoms (amenorrhea in women of reproductive age; low libido; and a lack of or atrophy of secondary sex characteristics, namely, pubic hair) in females and low LH levels in conjunction with low testosterone levels and symptoms (low libido; erectile dysfunction; and a lack of or atrophy of secondary sex characteristics, namely, pubic hair) in males. Diabetes insipidus was excluded based on such criteria as a serum osmolality of <295 mOsm/kg H_2_O concurrent with a urine osmolality of >600 mOsm/kg H_2_O while taking into consideration that this diagnosis can be only established based on a water-deprivation–vasopressin test.

Based on these homogeneous criteria, the selected patients were divided into 2 groups: Group 1: patients with no hormonal disorders (n = 37) and Group 2: patients with hormonal disorders (n = 6).

The statistical analysis was conducted with Statistica^®^ software (13.3), licensed for use by the Medical University of Warsaw. Statistical significance was determined with the use of tests suitable for the nature of data and the type of data distribution (verified with the Shapiro–Wilk W test) and variance homogeneity (verified with Brown–Forsythe’s test). Continuous variables were presented as means ± SD and medians and ranges, and the comparisons were made using either Student’s *t*-test for normally distributed variables with homogeneous variances or the Mann–Whitney U test for dependent variables showing departures from either normal distribution or variance homogeneity. Nominal variables were presented as absolute numbers and percentages and compared using either the chi-square test or Fisher’s exact test. A multivariate and univariate logistic regression models was used to determine the statistical significance of the evaluated variables in predicting hormonal disorders (adrenocortical insufficiency and hyperprolactinemia). The regression results were presented as odds ratios (OR) with 95% confidence intervals (95% CIs).

This study was approved by the ethics committee at the Medical University of Warsaw (approval No. KB/66/2022 of 16 May 2022). Each patient signed a written informed consent. This study was conducted according to the current standards, such as the Declaration of Helsinki.

## 3. Results

A total of six patients were found to have hormonal disorders. Four patients with hormonal disorders were found to have an empty sella (with the pituitary measuring less than 3 mm craniocaudally), and two were found to have a partial empty sella. The volume of suprasellar cistern herniation into the sella turcica exceeded 50% of sellar volume in all patients.

**Figure 2:**
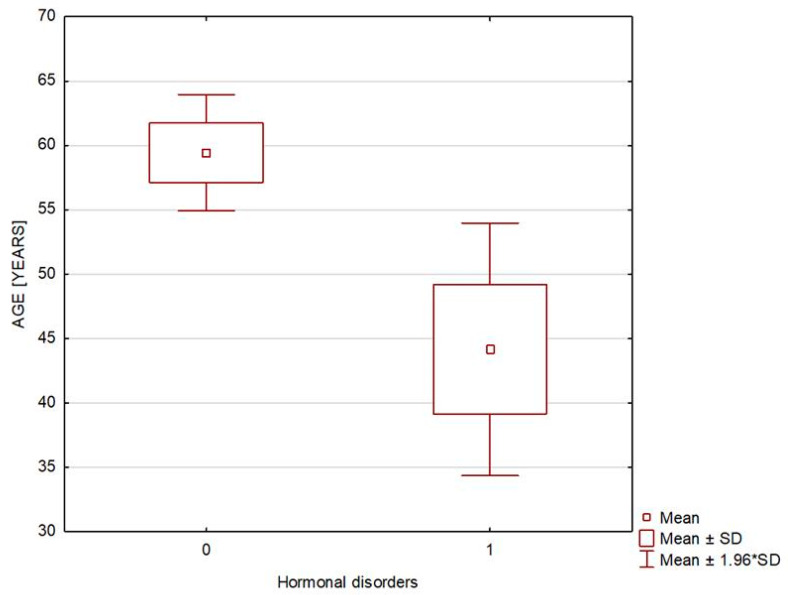
In figure, 0—patients without hormonal disorders (Group 1) and 1—patients with hormonal disorders (Group 2), broken down by age.

The patients from both study groups had good hormonal control, and the two groups differed only in electrolyte levels, which—nonetheless—stayed within normal limits. There was also a statistically significant difference in the waist circumference, body weight, and body mass index, all of which were higher in Group 1; however, there were no demonstrable differences in the rates of overweight or obesity. The two study groups differed in terms of patient age, with the hormonal disorder group significantly younger (Table 1 and Figure 2). Hyperprolactinemia was detected in four and secondary adrenocortical insufficiency in three of the patients with endocrine disorders. Two patients were receiving dopaminergic receptor agonists due to previously diagnosed hyperprolactinemia (associated with empty sella in one patient and with partial empty sella in the other). The two remaining patients, who were newly diagnosed with hyperprolactinemia (associated with empty sella in one patient and with partial empty sella in the other), were recommended to undergo outpatient follow-up assessments. All the patients with secondary adrenocortical insufficiency demonstrated an empty sella on MRI and were already receiving glucocorticoid replacement (Hydrocortisonum) due to the diagnosis having been established previously. None of the evaluated patients met the diagnostic criteria of growth hormone deficiency, hypogonadotropic hypogonadism, or diabetes insipidus (Table 2 and Table 3).

**Figure 3:**
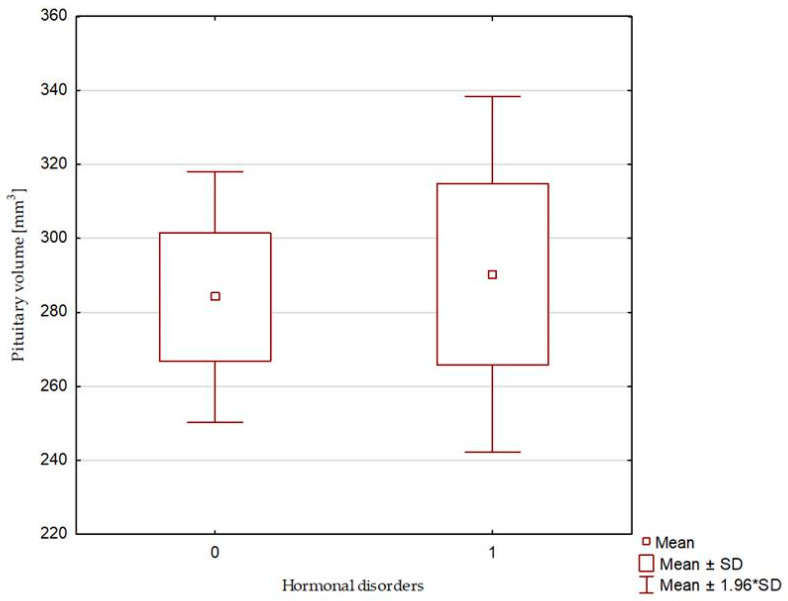
Pituitary volume. 0—patients without hormonal disorders (Group 1), and 1—patients with hormonal disorders (Group 2).

**Figure 4:**
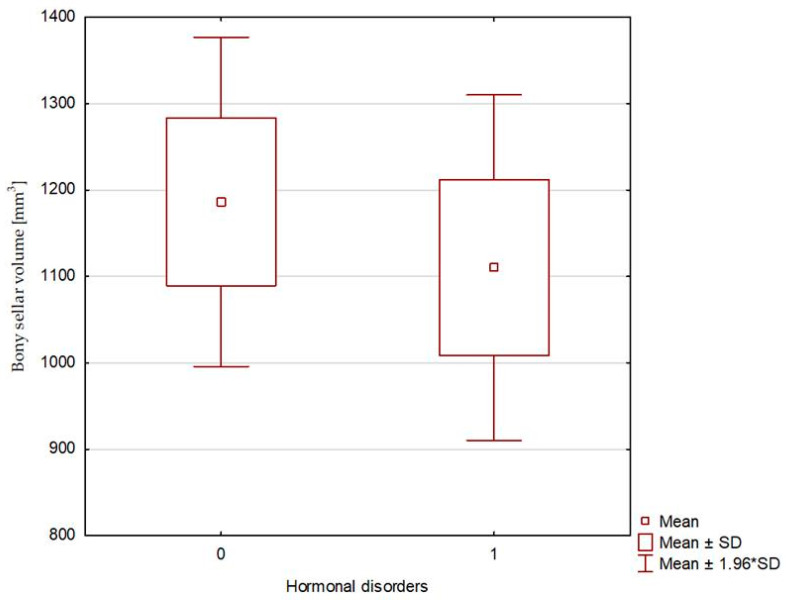
Sellar volume—calculated based on bony landmarks: 0—patients without hormonal disorders (Group 1), and 1—patients with hormonal disorders (Group 2).

**Figure 5:**
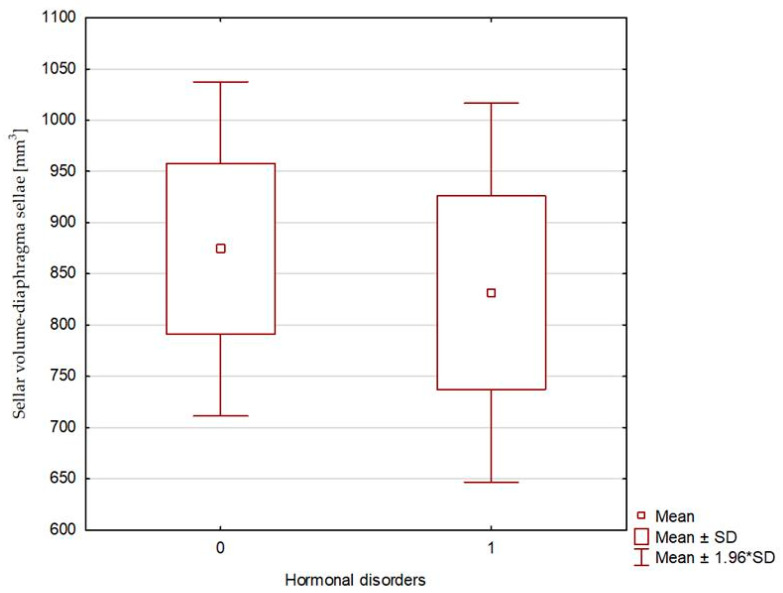
Sellar volume—calculated based on the location of the diaphragma sellae: 0—patients without hormonal disorders (Group 1), and 1—patients with hormonal disorders (Group 2).

**Figure 6:**
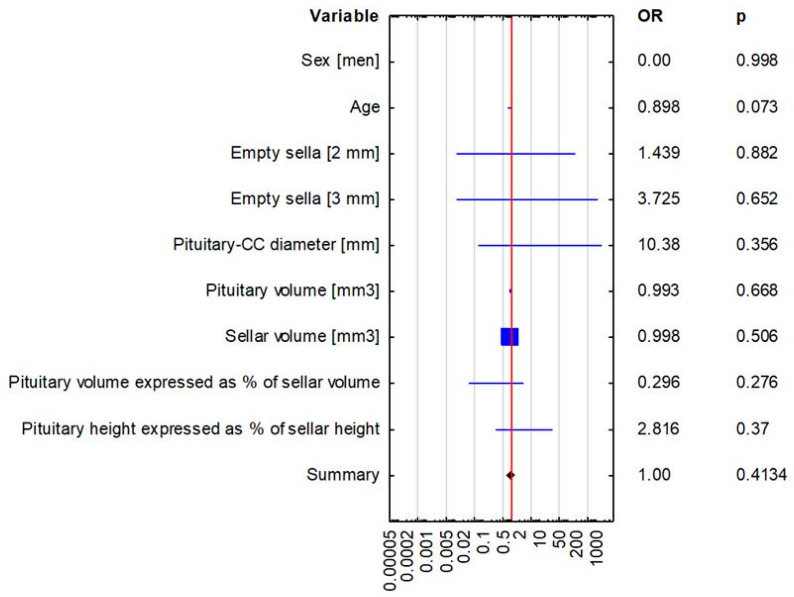
Multivariate logistic regression analysis in the assessment of predictors of hormonal disorders (adrenocortical insufficiency and hyperprolactinemia).

The compared study groups showed no differences in terms of the radiological parameters of either the pituitary or the sella turcica, or the relative pituitary-to-sellar volume or height (Table 4 and Figure 3, Figure 4 and Figure 5). Multivariate logistic regression showed no significant relationship between the evaluated radiological parameters and the development of hormonal disorders. The univariate logistic regression analysis revealed a significant relationship between age and the hormonal disorders (OR 0.916 [0.844–0.993]; *p* = 0.034), but this was not confirmed in the multivariate analysis (Table 5 and Figure 6). In our study, no patient exhibited evidence of posterior scleral flattening or critical transverse or sigmoid sinus stenosis; 11 patients showed enlarged cerebrospinal fluid spaces (dilated ventricles).

## 4. Discussion

Empty sella syndrome may produce neurological symptoms, such as headaches or visual disturbances, and endocrine manifestations, such as hyperprolactinemia or hypopituitarism [6,8]. These abnormalities usually prompt pituitary MRI, which may show evidence of empty or partial empty sella. However, in clinical practice, an empty or partial empty sella is more commonly detected incidentally on a scan performed for another reason on MRI and the patient is subsequently referred for further clinical evaluations and hormone tests [14,19,20]. In such cases, the radiological criteria for diagnosing an empty or partial empty sella and their correlation with hormonal disorders are of utmost importance.

Generally, an empty sella has been defined as herniation of the subarachnoid space into the sella turcica occupying more than 50% of its volume, which leads to pituitary flattening to less than 3 mm craniocaudally, whereas a partial empty sella has been characterized by the craniocaudal diameter of the pituitary being equal to or greater than 3 mm, with the cerebrospinal fluid occupying less than 50% of sellar volume [5,13,14,15]. Some authors have adopted the cutoff craniocaudal diameter of the pituitary to be 2 mm [9,15]. Moreover, some authors consider only either the criterion of pituitary craniocaudal diameter or the percentage of the sella occupied by the suprasellar cistern, with the latter most commonly determined by a cutoff of 50%, but other cutoff percentages, for example, 60%, have also been reported [14]. Depending on the pituitary height in relation to the sellar height measured in midsagittal T1-weighted images, the following grades of pituitary height loss have been defined: I—no pituitary height loss, II—mild loss of pituitary height by <1/3 of sellar height, III—moderate loss of pituitary height (between 1/3 and 2/3 of sellar height), IV—severe loss of pituitary height of more than 2/3 of sellar height, and V—total empty sella [21]. The radiological finding of an empty or a partial empty sella may be a manifestation of idiopathic intracranial hypertension. Other manifestations include flattening of the posterior scleral surface, transverse or sigmoid sinus stenosis, optic nerve sheath dilation, and optic nerve tortuosity [17]. The etiology of idiopathic increase in cerebrospinal fluid pressure above 250 mmHg H_2_O is unknown; however, risk factors of this condition are similar to those of empty or partial empty sella, which are obesity, the female sex, and reproductive age [22,23]. One of the suspected risk factors for idiopathic intracranial hypertension in patients with obesity is hyperaldosteronism. Aldosterone is known to increase the activity of the sodium–potassium pump that transports sodium ions from the cytoplasm of choroid epithelial cells into the cerebrospinal fluid, which causes the cerebrospinal fluid to experience an influx of sodium ions and, consequently, to increase in volume [22,23,24]. Another known effect of hyperaldosteronism is a drop in serum potassium levels. Our patients with empty and partial empty sella and endocrine disorders showed low normal serum potassium levels, which may be associated either with aldosterone levels in these patients or with the dose of hydrocortisone used as glucocorticoid replacement due to adrenocortical insufficiency. Hormonal disorders are not only known to result from empty or partial empty sella but also to be a risk factor for its development via their effects on intracranial pressure [17,25,26]. Examples of hormones with this potential effect are estrogens, which reduce aquaporin expression in hepatocytes and may theoretically exert a similar effect on the permeability of nervous system cells and increasing intracranial pressure [7,25,26,27]. Young women are known to have high estrogen levels, which is consistent with the characteristics of our study group with endocrine disorders, who comprised only women—young and overweight—although less overweight than patients without hormonal disorders.

Hormonal disorders were detected in six of the patients taking part in our study. Four were diagnosed with hyperprolactinemia and three with secondary adrenocortical insufficiency. Hyperprolactinemia is the most common endocrine disorder in patients with empty (or partial empty) sella syndrome, affecting 10–37.5% of patients with this diagnosis. Hyperprolactinemia may be associated with pituitary stalk compression and inhibition of hypothalamic dopamine release, while dopamine acts as a prolactostatin [7,28,29]. Adrenocortical insufficiency may affect up to 37.5% of patients, being more common in patients with empty sella than in those with partial empty sella. The pathogenesis of corticotropic cell dysfunction may be associated with their location in the anterior pituitary, which seems to make them more prone to compression by the suprasellar cistern [30,31].

The two evaluated groups did not differ significantly either in terms of an AP, an RL, or a CC diameter of the pituitary or in terms of pituitary volume. Similarly, there were no inter-group differences in terms of sellar dimensions or volume. The evaluated groups showed no differences in the relative pituitary-to-sellar volume, which determines the degree to which the sella is filled by the suprasellar cistern.

The multivariate logistic regression analysis conducted in our study showed no evidence to support that pituitary or sellar volume or any other radiographic parameters may be independent risk factors of hormonal disorders in patients with an empty or partial empty sella. The univariate logistic regression analysis revealed the patient’s age to be a risk factor of hormonal disorders, with younger patients with an empty or partial empty sella being at a significantly greater risk of developing hormonal disorders.

Similar conclusions regarding pituitary volume have been presented by other authors. A study evaluating the pituitary volume in the elderly showed no relationship between hormonal disorders and either empty sella or pituitary volume [32]. Another study showed age- and sex-related differences in pituitary volume between patients with empty and partial empty sella and patients without such a diagnosis, with an empty or partial empty sella associated with a more rapid loss of pituitary volume with increasing age. However, there was no demonstrable relationship between hormonal disorders and pituitary volume [33]. Pituitary volume is known to change with age and as a result of specific clinical situations associated with pituitary hormone secretion, such as pregnancy or menopause in women [33,34,35]. Generally, a normal pituitary measures up to 12 mm in width, 8 mm in the AP diameter, and 9 mm in height [36]. More specifically, normal pituitary height is up to 6 mm in children under 12 years old, up to 10 mm in adolescents, up to 9 mm in young adult women, up to 8 mm in young adult men, and up to 12 mm during pregnancy. After the age of 50 years, pituitary dimensions usually diminish; however, in the perimenopausal period, they may increase, although not to the extent observed during pregnancy [36]. The adopted dimensions of a normal sella turcica are 15 mm in length and 12 mm in depth, which nonetheless has no effect on hormonal disorders [37,38,39]. A different source provides the following morphometric parameters of the sella turcica—anterior height 6.71 mm (SE = 0.52); posterior height 6.93 (SE = 0.22); overall length 9.06 mm (SE = 0.15), specifically 8.94 mm (SE = 0.22) in females and 9.19 mm (SE = 0.26) in males; overall width 9.74 mm (SE = 0.31), specifically 9.78 mm (SE = 0.39) in females and 9.51 mm (SE = 0.4) in males; overall depth 8.00 mm (SE = 0.13); and overall volume 845.8 mm^3.^ (SE = 0.5) [1]. According to these data, the sellae turcicae in our patients were enlarged, especially when bony sella dimensions are considered. We calculated the pituitary and sellar volume with the use of a simplified ellipsoid formula, since it is the most readily accessible one, particularly in everyday clinical practice. At the same time, we took into consideration reports on normal pituitary glands that stated that this method of calculating pituitary volume may yield underestimated results in comparison with stereological approaches, such as the point-counting method and the planimetric method; we also took into consideration other reports, which showed planimetric and linear methods to yield overestimated results [35,40].

A detailed assessment of the pituitary gland is an important part of every MRI examination of the central nervous system. The dimensions and volume not only of the pituitary but also of the sella turcica are helpful in diagnosing and assessing patients with empty or partial empty sella but do not conclusively determine hormonal disorders. In light of the above, the currently used, simple definitions of empty and partial empty sella, where the diagnostic criteria are pituitary size and the percentage of sellar volume occupied by the suprasellar cistern, seem to be still valid and highly reproducible. Due to the fact that hormonal imbalances have been detected in patients with empty and partial empty sella, this group of patients may require endocrine testing. It is worth noting that younger patients with empty or partial empty sella are at an increased risk of developing hormonal disorders, which should be considered in planning hormone level tests.

One limitation of our study is a small sample size; however, the study population was selected from a larger population, in which the evaluated condition is uncommon.

In the future, prospective multicenter studies should be carried out on a large group of patients newly diagnosed with empty or partial empty sella, with long follow-up periods, calculation of pituitary volume by different methods, and potential risk factor assessment, including assessment of the renin–angiotensin–aldosterone system.

## 5. Conclusions

Although radiologic parameters of the pituitary and sella turcica help assess and diagnose patients with empty or partial empty sella, they do not conclusively determine hormonal disorders. Younger patients with empty or partial empty sella seem (at least in the univariate analysis) to have a higher odds ratio for developing hormonal disorders, which should be considered while planning hormone level tests. These findings suggest that radiological parameters alone are insufficient to predict hormonal dysfunction in patients with empty or partial empty sella. However, younger patients may be at higher risk, warranting closer hormonal monitoring.

## Figures and Tables

**Table 1:** Clinical characteristics. Group 1: patients without hormonal disorders (n = 37); Group 2: patients with hormonal disorders (n = 6).

Variable	Patients Without Hormonal Disorders n = 37	Patients with Hormonal Disorders n = 6	*p*-Value
Age [years]	59.432 ± 14.041	44.167 ± 12.254	0.021
Sex [M]	9 (2.324%)	0 (0%)	0.187
Body weight [kg]	79.697 ± 13.462	67.583 ± 10.414	0.034
BMI [kg/m^2^]	28.096 ± 4.567	25.45 ± 3.169	0.045
Overweight and obesity	27 (72.973%)	3 (50%)	0.271
Waist circumference [cm]	95.125 ± 11.284	79.833 ± 15.943	0.022
Arterial hypertension	16 (43.243%)	1 (16.667%)	0.16

BMI—body mass index.

**Table 2:** Group 1 and Group 2 characteristics; Group 1: patients without hormonal disorders (n = 37), and Group 2: patients with hormonal disorders (n = 6).

Variable	Patients Without Hormonal Disorders n = 37		Patients with Hormonal Disorders n = 6		*p*-Value
	Median (Min–Max)	Mean ± SD	Median (Min–Max)	Mean ± SD	
Specific gravity of urine [g/mL]	1.016 (1.007–1.04)	1.017 ± 0.007	1.013 (1.006–1.028)	1.016 ± 0.009	0.663
Serum sodium [mmol/L]	142 (128–145)	141 ± 3.04	137.50 (136–141)	138 ± 1.79	0.005
Serum potassium [mmol/L]	4.55 (3.78–5.39)	4.54 ± 0.39	4.13 (3.69–4.49)	4.12 ± 0.29	0.016
Serum osmolality [mOsm/kg]	291 (278–313)	290.87 ± 6.79	287 (282–293)	287.33 ± 3.67	0.171
Urine osmolality [mOsm/kg]	539.50 (251–956)	571.75 ± 203.21	440.5 (258–957)	558.33 ± 304.79	0.787
TSH [µIU/mL]	1.95 (0.27–4.08)	1.959 ± 0.97	2.02 (0.95–2.82)	1.90 ± 0.74	0.899
fT3 [pg/mL]	3.06 (2.16–3.82)	3.10 ± 0.39	2.75 (2.34–3.58)	2.83 ± 0.43	0.109
fT4 [ng/dL]	1.26 (1.02–1.70)	1.27 ± 0.162	1.10 (0.90–1.33)	1.11 ± 0.168	0.081
Prolactin [ng/mL]	11.00 (4.9–20.8)	11.86 ± 5.21	15.15 (3.20–52.60)	22.08 ± 18.69	0.326
Cortisol at 8 a.m. [µg/dL]	14.30 (6.1–24.5)	13.68 ± 3.63	11.6 (8.90–24.30)	14.73 ± 6.56	0.746
ACTH at 8 a.m. [pg/mL]	22.5 (7.3–87.2)	25.82 ± 16.29	15.8 (2.30–28.90)	16.08 ± 9.04	0.136
Growth hormone [ng/mL]	0.44 (0.05–17.10)	1.32 ± 2.99	0.45 (0.17–8.96)	2.17 ± 3.47	0.472
IGF-1 [ng/mL]	135.50 (61.33–253.50)	140.84 ± 46.41	188.70 (82.65–350.80)	195.09 ± 94.43	0.166

ACTH—adrenocorticotropic hormone; fT3—free triiodothyronine; fT4—free thyroxine; IGF-1—insulin-like growth factor; SD – standard deviation, TSH—thyroid-stimulating hormone.

**Table 3:** Hormone levels in patients with endocrine disorders.

Variable	Mean	SD	Median	Minimum	Maximum
ACTH [pg/mL] (n = 6)	16.067	9.041	15.800	2.300	28.900
ACTH [pg/mL] in patients with secondary adrenocortical insufficiency on glucocorticoid replacement with Hydrocortisonum (n = 3)	10.667	7.786	12.000	2.300	17.700
Serum cortisol [µg/dL]	14.733	6.563	11.600	8.900	24.300
Serum cortisol [µg/dL] in patients with secondary adrenocortical insufficiency on glucocorticoid replacement with Hydrocortisonum (n = 3)	18.300	8.24	21.700	8.900	24.300
Serum prolactin [ng/mL] (n = 6)	22.083	18.691	15.150	3.200	52.600
Serum prolactin [ng/mL] in patients with hyperprolactinemia (n = 4)	26.700	22.154	25.500	3.200	52.600
Serum prolactin [ng/mL] in patients with hyperprolactinemia receiving dopamine receptor agonists (n = 2)	8.800	7.919	8.800	3.200	14.400
Serum prolactin [ng/mL] in patients with hyperprolactinemia receiving no dopamine receptor agonists (n = 2)	44.600	11.314	44.600	36.600	52.600
TSH [µIU/mL]	1.904	0.741	2.020	0.952	2.820
fT3 [pg/mL]	2.833	0.426	2.745	2.340	3.580
fT4 [ng/dL]	1.110	0.168	1.100	0.900	1.330
Growth hormone [ng/mL]	2.168	3.465	0.450	0.170	8.960
IGF-1 [ng/mL]	195.092	94.434	188.700	82.650	350.800

ACTH—adrenocorticotropic hormone; fT3—free triiodothyronine; fT4—free thyroxine; IGF-1—insulin-like growth factor; SD-standard deviation, TSH—thyroid-stimulating hormone.

**Table 4:** Radiologic characteristics of the patients. Group 1: patients without hormonal disorders (n = 37), and Group 2: patients with hormonal disorders (n = 6).

Variable	Patients Without Hormonal Disorders n = 37		Patients with Hormonal Disorders n = 6		*p*-Value
	Median (Min–Max)	Mean ± SD	Median (Min–Max)	Mean ± SD	
Pituitary AP diameter [mm]	12.00 (7.50–17.10)	12.40 ± 2.03	12.00(10.00–13.30)	12.00 ± 1.38	0.659
Pituitary RL diameter [mm]	16.00 (5.00–25.00)	15.95 ± 3.27	16.25 (14.40–20.00)	16.80 ± 2.39	0.516
Pituitary CC diameter [mm]	2.80 (0.80–5.00)	2.97 ± 1.06	2.90 (1.40–4.50)	3.05 ± 1.093	0.752
Pituitary volume [mm^3^]	276.12 (62.40–536.30)	284.14 ± 105.12	299.55 (194.194–368.00)	290.29 ± 60.05	0.739
Sella turcica—AP diameter [mm]	12.00 (7.50–17.10)	12.51 ± 2.08	12.50 (10.00–14.30)	12.38 ± 1.15	0.902
Sella turcica—RL diameter [mm]	16.00 (5.00–25.00)	15.95 ± 3.27	16.25 (14.40–20.00)	16.80 ± 2.39	0.516
Sella turcica—bony—CC diameter [mm]	10.40 (7.30–17.40)	11.25 ± 2.53	10.40 (9.50–13.30)	10.68 ± 1.43	0.833
Sella turcica—diaphragma sellae—CC diameter [mm]	7.40 (5.50–15.70)	8.20 ± 2.46	7.90 (6.50–9.70)	7.97 ± 1.26	0.699
Bony sellar volume [mm^3^]	976.19 (140.63–1683.71)	1186.41 ± 591.43	1099.03 (792.00–1498.07)	1110.28 ± 249,81	0.661
Sellar volume—diaphragma sellae [mm^3^]	665.63 (116.25–2316.99)	874.48 ± 506.25	769.97 (597.60–1248.39)	831.69 ± 231.16	0.451
Pituitary volume expressed as a percentage of sellar volume [%]	28.79 (6.02–66.67)	28.15 ± 12.93	27.25 (12.96–40.91)	28.14 ± 10.91	0.986
Pituitary height expressed as a percentage of bony sella height [%]	28.79 (6.02–66.67)	28.35 ± 12.94	28.95 (12.96–40.91)	28.93 ± 10.74	0.875
Pituitary height expressed as a percentage of sella height—diaphragma sellae [%]	39.79 (9.09–80.65)	40.08 ± 19.25	41.43 (15.56–54.22)	39.31 ± 14.84	0.986
Right optic nerve sheath diameter [mm]	4.65 (3.80–5.80)	4.71 ± 0.44	4.75 (4.40–5.30)	4.77 ± 0.33	0.793
Left optic nerve sheath diameter [mm]	4.60 (3.70–6.30)	4.56 ± 0.51	4.5 (3.90–5.30)	4.57 ± 0.47	0.930
VARIABLE	N (%)		N (%)		*p*-value
Empty sella—with the pituitary CC diameter less than 2 mm	8 (21.62%)		1 (16.67%)		0.804
Empty sella—with the pituitary CC diameter less than 3 mm	19 (51.35%)		4 (66.67%)		0.503
Pituitary stalk displacement	21 (56.76%)		3 (50.00%)		0.757
Enlarged cerebrospinal fluid spaces	10 (27.03%)		1 (16.67%)		0.589

**Table 5:** Multivariate and univariate logistic regression in the assessment of the predictors of hormonal disorders (adrenocortical insufficiency and hyperprolactinemia).

Variable	Hormonal Disorders OR (95% CI) *p*-Value	
	Multivariate Logistic Regression	Univariate Logistic Regression
Sex	0.00 (0.00–0.00), *p* = 0.998	0.00 (0.00–0.00), *p* = 0.997
Age	0.898 (0.798–1.01), *p* = 0.073	0.916 (0.844–0.993), *p* = 0.034
Empty sella [2 mm]	1.439 (0.008–297.121), *p* = 0.882	0.725 (0.074–7.126), *p* = 0.783
Empty sella [3 mm]	3.725 (0.012–1131.502), *p* = 0.652	1.895 (0.308–11.644), *p* = 0.49
Pituitary—craniocaudal diameter [mm]	10.380 (0.072–1495.711), *p* = 0.356	1.073 (0.469–2.454), *p* = 0.8668
Pituitary volume [mm^3^]	0.993 (0.964–1.024), *p* = 0.668	1.001 (0.992–1.009), *p* = 0.887
Sellar volume [mm^3^]	0.998 (0.992–1.004), *p* = 0.506	1 (0.998–1.001), *p* = 0.7531
Pituitary volume expressed as percentage of sellar volume(bony sella)	0.296 (0.033–2.639), *p* = 0.276	1 (0.933–1.072), *p* = 0.999
Pituitary height expressed as percentage of sellar height (bony sella)	2.816 (0.293–27.105), *p* = 0.37	1.004 (0.937–1.076), *p* = 0.916

OR, presented as OR (95% CI—+ 95% CI), calculated with multivariate logistic regression analysis; crude OR values are optionally adjusted for age and sex.

## Data Availability

The datasets generated during and/or analyzed during the present study are available from the corresponding author on reasonable request.
